# Immune-Checkpoint Blockade and Active Immunotherapy for Glioma

**DOI:** 10.3390/cancers5041379

**Published:** 2013-11-01

**Authors:** Brian J. Ahn, Ian F. Pollack, Hideho Okada

**Affiliations:** 1 Department of Immunology, University of Pittsburgh School of Medicine, Pittsburgh, PA 15213, USA; E-Mail: bra14@pitt.edu; 2 Brain Tumor Program, University of Pittsburgh Cancer Institute, Pittsburgh, PA 15213, USA; E-Mail: Ian.Pollack@chp.edu; 3 Department of Neurological Surgery, University of Pittsburgh School of Medicine, Pittsburgh, PA 15213, USA; 4 Department of Surgery, University of Pittsburgh School of Medicine, Pittsburgh, PA 15213, USA

**Keywords:** glioma, cancer vaccines, tumor immunity, T cells, tumor microenvironment, cancer immunotherapy

## Abstract

Cancer immunotherapy has made tremendous progress, including promising results in patients with malignant gliomas. Nonetheless, the immunological microenvironment of the brain and tumors arising therein is still believed to be suboptimal for sufficient antitumor immune responses for a variety of reasons, including the operation of “immune-checkpoint” mechanisms. While these mechanisms prevent autoimmunity in physiological conditions, malignant tumors, including brain tumors, actively employ these mechanisms to evade from immunological attacks. Development of agents designed to unblock these checkpoint steps is currently one of the most active areas of cancer research. In this review, we summarize recent progresses in the field of brain tumor immunology with particular foci in the area of immune-checkpoint mechanisms and development of active immunotherapy strategies. In the last decade, a number of specific monoclonal antibodies designed to block immune-checkpoint mechanisms have been developed and show efficacy in other cancers, such as melanoma. On the other hand, active immunotherapy approaches, such as vaccines, have shown encouraging outcomes. We believe that development of effective immunotherapy approaches should ultimately integrate those checkpoint-blockade agents to enhance the efficacy of therapeutic approaches. With these agents available, it is going to be quite an exciting time in the field. The eventual success of immunotherapies for brain tumors will be dependent upon not only an in-depth understanding of immunology behind the brain and brain tumors, but also collaboration and teamwork for the development of novel trials that address multiple layers of immunological challenges in gliomas.

## 1. Introduction

The main premise of cancer immunotherapy is to harness the patient’s own immune system to attack malignant tumor cells. This area of research has made tremendous progress, illustrated by the United States Food and Drug Administration (FDA) approval of a monoclonal antibody (mAb), ipilimumab, for treatment of metastatic melanoma [1,2] and a vaccine for prostate cancers as the first vaccine against non-viral cancers [3]. For central nervous system (CNS) cancers, early phase immunotherapy trials showed encouraging outcomes. Nonetheless, the immunological microenvironment of the CNS and tumors arising in the CNS is still believed to be suboptimal for sufficient antitumor immune responses for a variety of reasons, including the operation of “immune-checkpoint” mechanisms. These are mechanisms that inhibit the function of cytotoxic immune responses and induce immunological tolerance. While these mechanisms prevent autoimmunity in physiological conditions, malignant tumors, including brain tumors, actively employ these mechanisms to evade from immunological attacks. Development of agents designed to unblock these checkpoint steps is currently one of the most active areas of cancer research. In this review, we first discuss recent advances in our understanding of drug-development against immune-checkpoint mechanisms. We also review advances in active immunotherapy strategies for glioma, with an emphasis on lessons learned from recent clinical trials. 

## 2. Immunosuppression by Gliomas

The microenvironment of gliomas, including that of glioblastoma (GBM), is known to produce immunosuppressive molecules and recruit immunosuppressive leukocytes.

### 2.1. Immunosuppressive Factors

Gliomas have been shown to synthesize and secrete multiple factors that are capable of inhibiting anti-tumor immune responsiveness [4,5]. The list of these factors have expanded and further characterized over the last several years. It is also important to develop strategies to block the function of these molecules, thereby promoting anti-tumor immune responses. In this section, we update and summarize each of these factors that appear to be relevant to gliomas.

#### 2.1.1. Transforming Growth Factor β (TGF-β)

TGF-β is one of the most potent immunosuppressive cytokines; its biological effects are multiple and complex [6]. Specifically, for immune cells, they include the inhibition of (1) antigen-presenting cell (APC) maturation (2) functions of APCs, (3) T-cell activation, and (4) their differentiation towards effector cells. Recent studies have shown that TGF-β is up-regulated in glioma cell subpopulations that are resistant to the cytotoxic effects of allogeneic cytotoxic T-cells (CTLs) [7,8]. 

TGF-β can abrogate immune rejection by inhibiting natural killer (NK) cells function. NKG2D is an NK-cell activating receptor, but GBM exhibited decreased NKG2D-mediated tumor recognition and lysis due to TGF-β promoted down-regulation of NKG2D ligands on glioma cells [9] and NKG2D on NK and T cells [10]. Inhibition of TGF-β by interfering RNA [11] or soluble TGF-β receptor [12,13] resulted in improved NKG2D-mediated immune response and prolonged survival in mouse models. 

TGF-β inhibition can potentially bolster vaccine efficacy for more robust anti-tumor immune surveillance. Both TGF-β anti-sense oligonucleotides [14] and systemic blockade of TGF-β by specific monoclonal antibody (mAb) [8] enhanced the efficacy of glioma-specific vaccines and improved survival. Although in both studies, blockade of TGF-β alone had no significant impacts on prolonging the survival of glioma-bearing mice.

In addition to immune cells, TGF-β can have direct effects on glioma cells and glioma stem cell populations, and can promote tumor development independent of immune suppression. Recent studies have examined the impact of TGF-β on glioma-initiating cells (GIC). Targeting TGF-β receptors may specifically target GIC [15], where TGF-β signaling has been linked with tumor propagating properties of GIC, such as invasion [16], radiation resistance [17], self-renewal [18], and tumorogenecity [19]. 

A recent study demonstrated that TGF-β stimulation induces microRNA-182 expression, thereby aberrantly activating NF-κB signaling in GBM cells [20], clarifying a critical point of cross-talk between molecular signaling pathways. These findings provide a greater understanding of the complex interplay between signaling pathways in cancer that may ultimately prove useful in the development of synergistic targeting approaches. 

Preclinical data suggest TGF-β inhibition as an attractive target for treatment of human gliomas for its diverse effects on multiple cell types, which culminate in promoting glioma development. TGF-β-targeting antisense oligonucleotides showed improved survival compared to historical controls of recurrent and refractory high-grade glioma in phase I/II, but the phase III trials were terminated due to the inclusion of temozolomide (TMZ) in the standard-of-care in GBM therapy and resulting lack of patient accrual. Currently, investigations into oral TGF-β receptor 1 inhibitors and TGF-β blocking antibody are underway in phase II trials [21].

#### 2.1.2. Interleukin 10 (IL-10)

IL-10 is primarily produced by monocytes and, to a lesser extent, type 2 T helper (Th2) lymphocytes, mastocytes, regulatory T cells (Tregs), and in a certain subset of activated T cells and B cells [22]. IL-10 can be produced by monocytes upon programmed death (PD)-1 triggering in these cells [23]. Like TGF-β, this cytokine has pleiotropic effects in immunoregulation and inflammation. IL-10 down-regulates the expression of Th1 cytokines, MHC class II antigens, and costimulatory molecules on APCs. The expression levels of IL-10 in glioma tissue correlate with glioma grade as well as a degree of brain invasiveness [24,25]. 

#### 2.1.3. Prostaglandin E_2_ (PGE_2_)

Cyclooxygenase-2 (COX-2) catalyzes the conversion of arachidonic acid to prostaglandins. COX-2 and its product PGE_2_ are known to promote tumor growth, angiogenesis, and invasion of cancer cells [26,27]. COX-2 and PGE_2_ also promote immune escape of tumors through a variety of mechanisms, including induction Tregs [28], suppression of T-helper (Th)1 cells [29,30], and recruitment of Th2 cells [31,32,33]. PGE_2_ induces the differentiation of Gr1^+^CD11b^+^ myeloid-derived suppressor cells (MDSCs) from bone marrow stem cells [34]. Furthermore, PGE_2_ produced by the tumor induces arginase 1 and cationic amino acid transporter (CAT)-2B in MDSCs, both of which deplete arginine from the tumor microenvironment and impair T-cell function [35,36]. We recently demonstrated in our *de novo* glioma model that treatment with the COX-2 inhibitors acetylsalicylic acid (ASA) or celecoxib inhibited systemic PGE₂ production and delayed glioma development [37]. ASA treatment also reduced monocyte chemoattracting protein (MCP)-1, also known as CCL2 (C-C motif ligand 2), in the glioma as well as the number of MDSCs in both bone marrow and the glioma. Both ASA-treated and *Cox2^−^*^/−^ mice demonstrated a reduction in MDSCs and their CCL2-mediated accumulation in the glioma. Conversely, *Cox2^−^*^/−^ mice and ASA-treated wild-type mice displayed enhanced expression of CXCL10 (C-X-C motif chemokine 10) and tumor-infiltration of CTLs [37]. 

#### 2.1.4. CCL2 (Also Known as Macrophage Chemoattractive Protein 1; MCP-1)

CCL2 is a chemokine secreted by a variety of glioma cell lines and expressed in GBMs [38,39]. In addition to its angiogenetic effects [40], it is associated with recruitment of immunosuppressive leukocytes, such as MDSC and regularory T-cells (Tregs) [37,41,42,43]. Systemic blockade of CCL2 exhibited reduced infiltration of MDSC and tumor-associated macrophages (TAM) into the glioma site and prolonged survival in murine models. Concurrent chemotherapy with TMZ further enhanced the survival [44]. TMZ treatment of GBM cells *in vitro* demonstrated decreased CCL2 production and subsequently reduced migration of Tregs [42], suggesting a possible additive effect of TMZ and CCL2 blockade strategies. 

#### 2.1.5. Fas Receptor (FasR)/Ligand (FasL)

The Fas receptor is a death receptor on the surface of cells that leads to apoptosis [45,46,47]. Malignant gliomas express FasL, which induces apoptotic cell death of FasR-expressing adjacent immune cells infiltrating into tumors [47]. In addition, FasR expressed on glioma cells induces proinflammatory and angiogenic mediators, which in turn protect and support tumors growth [48]. 

Activation of Fas signaling in tumor cells may represent a possibly efficacious adjuvant to current anti-tumor therapy. Most treatments, such as some chemotherapies [49,50], indirectly target this pathway by up-regulating FasR, thereby promoting cell apoptosis. Additionally, some success has been demonstrated by directly activating this pathway. Treating gliomas with recombinant FasL and concurrent etoposide demonstrated increased tumor cell apoptosis of human GBM cells *in vitro* and improved symptom-free survival *in vivo* in mouse xenograft models [51]. The effect of FasL on T-cell functioning or apoptosis was not examined in this study, and further studies should examine any collateral effects of exogenous FasL on T-cell function. 

FasL knockdown with silencing RNA in rat glioma cell lines exhibited reduced tumor growth and increased CD3 T-cell infiltration *in vivo* [52]. This particular study utilized knock down of FasL in glioma cells prior to transplantation into their mouse model. Another examination utilized viral vectors to transduce glioma cells with FasL and Fas-associated death domain (FADD). These authors compared transduction efficacy when glioma cells were transduced prior to tumor transplantation (*ex vivo* transduction) or direct administration of the gene vector into established murine tumors (*in vivo* transduction), resulting in a 95% and 18% rate of cell transduction, respectively. Intracranial implantation of pre-transduced glioma cells resulted in better survival outcome when compared with viral vectors inoculated one week post-implantation of tumor cells, indicating that therapeutic efficacy is dependent on the viral spread and mode of viral vector administration. Concurrent treatment with TMZ or radiation therapy exhibited prolonged survival compared to FAS/FADD viral vector alone [53].

#### 2.1.6. Cytotoxic T-Lymphocyte Antigen-4 (CTLA-4)

CTLA-4 is a homologue of CD28, and expressed primarily on activated lymphocytes, where it competitively binds B7-1 and B7-2 due to its higher affinity for these molecules compared to CD28. Signaling through CTLA-4 decreases T cell responsiveness and activation [54]. Regulatory T cells constitutively express CTLA-4, where it is thought to play an important role in their suppressive capabilities [55].

The United States FDA recently approved an anti-CTLA-4 mAb ipilimumab for treatment of metastatic melanoma [1,2]. In preclinical mouse glioma models, systemic blockade of CTLA-4 exhibited increased survival [56]. This blockade was associated with increased systemic CD4 T cell counts, as well as reduced Treg function but not frequency. There was also no induction of CNS related autoimmunity, a major concern for this treatment from trials in other cancer types. Additionally, when used in combination with a glioma vaccine of irradiated glioma cell lines expressing granulocyte macrophage-colony stimulating factor (GM-CSF), there was improved survival and increased levels of IFN-γ production compared to treatment with CTLA-4 blocking mAb alone [57]. 

#### 2.1.7. B7-Homologue 1 (B7-H1); Programmed Death Ligand-1 (PD-L1)

The B7 family consists of co-stimulatory molecules that positively and negatively regulate immune responses. Among them, B7-homologue 1 (B7-H1), also known as programmed death ligand-1 (PD-L1), exerts immunosuppressive functions when interacting with its receptor PD-1 [58]. Glioma cells express B7-H1, which inhibits T-cell functions via suppression of cytokine production levels (IFN-γ, IL-2, and IL-10) and induction of apoptosis in tumor-specific T-cells [59]. GBMs often possess mutations in a tumor-suppressor gene, phosphatase and tensin homolog (PTEN), which are associated with up-regulation of B7-H1 [60]. Interestingly, IFN-γ treatment potentiated glioma immune evasion in PTEN-deficient glioma cells by promoting B7-H1 expression and T-cell apoptosis [61]. 

Targeting the PI3K/mTOR pathway with selective inhibitors was demonstrated to be a potential mechanism of reducing B7-H1 expression and improving T cell function [62]. Targeting this pathway with PD-1 blocking antibody was evaluated in conjunction with stereotactic radiotherapy in murine models of intracranial gliomas [63]. While the PD-1 blockade alone exhibited only a minimal increase in median survival, the combined therapy with stereotactic radiotherapy demonstrated a more drastic impact compared to either treatment alone. This improved survival was associated with increased CD8+ lymphocyte and decreased Treg infiltration into the tumor site. Currently, clinical trials are ongoing for multiple refractory and recurrent cancer types utilizing both anti-PD-1 [64] and anti-PD-L1 [65] blocking mAbs, and although early in their investigations, these antibodies are showing promising results.

### 2.2. Immunosuppressive Leukocytes

A large number of observations suggest that certain types of immune cells in the tumor microenvironment (TME) are not innocent bystanders at brain tumor sites, but may actually promote tumor development and progression [66,67,68]. MDSCs and Tregs may affect these processes via their ability to express a large variety of factors, including immunoregulatory cytokines and cell surface molecules, which can suppress T-cell activation, proliferation, trafficking and effector function. These cytokines may be secreted not only by inflammatory cells, but also by the tumor cells and stroma cells, together establishing a local environment that significantly promotes brain tumor growth. These cell types are a potential target for improving efficacy of immunotherapies and prolonging survival of patients with malignant gliomas (Table 1). In glioma models and patients, Tregs have been the predominant targets, and research into modulating MDSC populations is still early in its inception for treatment of malignant gliomas.

#### 2.2.1. Myeloid-Derived Suppressor Cells (MDSCs)

Expanded populations of immature myeloid cells have been demonstrated in essentially all cancer types [66]. Pathological conditions such as cancer can halt normal maturation of these cells, promote expansion of these immature myeloid cells, and induce suppressive effectors [66,69,70]. These cells possess immature phenotype and morphology, and can promote tumor development via T-cell suppression dependent and independent mechanisms. 

In mice, these immature myeloid cells are routinely identified by the expression of CD11b and Gr1. Neutrophilic (G-MDSC) and monocytic (M-MDSC) subtypes can be further stratified by expression of Ly6G and Ly6C, respectively. These cells have demonstrated T-cell suppression via various effects such as arginase1 [71], inducible nitric oxide synthase (iNOS) [71], reactive oxygen species (ROS) [72], depletion of cysteine [73], among others. They have also been demonstrated to promote tumor angiogenesis, invasion, proliferation, and metastasis. In murine models, selective depletion of these cells has shown reduced tumor growth and improved survival [37].

In human cancers, these cells are identified by expression of CD11b and CD33 and by lack of expression of human leukocyte antigen (HLA)-DR with similar granulocytic and monocytic subtypes, differentiated by expression of CD15 and CD14, respectively [74]. The accumulation of these cells and their subtypes, differ depending on the cancer type. In a recent phase II study of peptide-based vaccines in HLA-A*02+ patients with advanced renal cell carcinoma, the numbers of CD14+HLA-DR−/low and CD11b+CD14−CD15+ MDSC were significantly negatively associated with overall survival [75].

**Table 1:** Agents targeting immunosuppressive cells.

Agent (+immunotherapy)	Study findings	Reference
**Agents targeting Tregs**		
Anti-CD25 (IL-2Rα) Mab		
mAb+mRNA-loaded DC	Decrease in Treg number/frequency; Reduced Treg suppression in mice with mAb alone	[76]
	Improved functions of CD8 T cells and tumor cell lysis	
	Improved survival with anti-CD25 mAb alone, and further improvement with DC vaccine	
mAb+peptide-loaded DC	Anti-CD25mAb tx exhibited decreased Treg for >3 weeks in mice	[77]
	Prophylactic but not therapeutic effects against glioma by DC vaccine alone	
	Combination with anti-CD25 mAb allows for improved therapeutic efficacy and survival	
	Increased IFNγ by T cells with vaccine+mAb	
	Anti-CD25 mAb alone improved survival in their murine model	[78]
	No persistent memory against re-challenged with tumor with anti-CD25 mAb alone	
	DC vaccine+ant-CD25 mAb improved survival after tumor re-challenge	
mAb+EGFR vIII vaccine	Depletion of Tregs after single mAb infusion, Decreased until day 122 post-infusion	[79]
	Increased ratio of effector to Treg after single infusion, Increased *in vitro* IFNγ by CD4 T cells	
	Inverse correlation between frequency of Treg and humoral response to EGFRvIII	
Temozolomide	Low dose, metronomic dose reduced Treg frequencies in spleen in rats	[80]
	*In vitro* culture with TMZ can reduce CCL2 production by glioma cells and reduce Treg recruitment	[42]
**MDSC-targeting agents**		
COX-2 Inhibitors	Celecoxib or acetylsalicylic acid treatment alone improved survival in murine models	[37]
	Decreased CCL2 production and reduced MDSC recruitment	
	Increased CXCL10, T cell infiltration and improved cytotoxic function at glioma site	
CCL2-neutralizing mAb	Reduced MDSC at tumor site in murine models	[44]
mAb + TMZ	Modest improvements in survival with neutralizing mAb alone	
	Improved survival with mAb + TMZ compared to TMZ alone	
Sunitinib		
Sunitinib + TMZ	Inhibition of *in vivo* tumor growth in murine model with Sunitinib alone	[81]
	Further inhibition of growth when Sunitinb + TMZ	
Sunitinib + Radiation	Suitinib alone showed a modest improvement of survival in mouse model	[82]
	High dose radiation + Sunitinib results in fatal toxicity	
	Low dose + Sunitinb exhibit a decrease in radiographic tumor volume but no change in survival	
Sunitinib + Gefitinib	No improvement in survival with Sunitinib alone; did not improve Gefitinib efficacy	[83]
Phase II Trials as single agent	No objective response in any pt; progression free survival: ~8 weeks	[84]
	No improvement in survival	[85]
	Tx in Bevacizumab resistant and naïve pts: no change in PFS in either cohort	[86]
ATRA	*In vitro* culture promoted differentiation of glioma stem cells	[87]
	*In vitro* culture reduced self renewal and proliferation of GBM stem cells	[88]
	Increased apoptosis of GBM stem cells	
PDE-5 Inhibitors	Vardanefil/Sidenefil increased permeability of tumor cells *in vivo*	[89]
	No change in survival with PDE-5 inhibition alone	
	Improved penetration by Adriamcyin and survival with Vardanefil + Adriamycin	

In human GBM, expanded populations of circulating CD15^+^CD33^+^HLA-DR^−^ and increased levels of plasma arginase 1 have been observed [90]. The impact of these suppressive cells require further elucidation such as their role in limiting anti-tumor immunity at the tumor site and prognostic significance on survival in patients with malignant gliomas.

In our preclinical study, neutralizing mAb against CCL2, a chemokine with MDSC attracting properties, reduced MDSC infiltration into gliomas in mice. When administered alone, there was a small but significant increase in survival. When used in combination with TMZ treatment, additional improvements in survival were observed compared to TMZ alone [44]. Additionally, we also demonstrated direct depletion of MDSC populations with anti-Gr1 antibodies improves survival of murine glioma models [37]. Our studies suggest the importance of targeting MDSC in the treatment of malignant gliomas.

Many MDSC modulating agents have been examined as a means to improve efficacy of immunotherapies in many cancer types. These agents can be broadly classified depending on their predominant mechanisms for reducing MDSC-mediated suppression into three groups: (1) promoting differentiation of MDSC; (2) decreasing MDSC numbers; (3) reducing MDSC function. A more comprehensive review of targeting MDSC in all tumor types can be found in a recent review [91]. Many of these therapies have already been examined in treatment of gliomas, but no studies have examined the changes to immune function and the effects on concurrent immunotherapy. 

#### 2.2.2. Inducing Differentiation of MDSC

All-trans retinoic acid [28], a derivative of vitamin A, can promote differentiation of MDSC via glutathione-dependent reduction of ROS [92]. Treatment with ATRA reduced MDSC populations, increased functions of Th1 T cell response, and improved immune responses against tumor antigens in patients with small cell lung cancer [93] and similarly in renal cell carcinoma [94]. Currently, in glioma, *in vitro* experiments have demonstrated that ATRA reduced stem cell characteristics such as self-renewal and proliferation [87,88] but its impact on MDSC populations and overall survival remain to be investigated. Vitamin D_3_ has also been demonstrated to promote myeloid progenitor differentiation and reduction of immunosuppression in head and neck squamous cell carcinomas. With treatment, patients also exhibited increased plasma IFN-γ levels [95].

#### 2.2.3. Reducing MDSC Numbers

Sunitinib is an oral, small-molecule, multi-targeted receptor tyrosine kinase (RTK) inhibitor that was approved by the FDA for the treatment of renal cell carcinoma (RCC) and imatinib-resistant gastrointestinal stromal tumor. Molecular targets for sunitinib include all receptors for platelet-derived growth factor, vascular endothelial growth factor receptors and CD117, all of which play a role in both tumor angiogenesis and tumor cell proliferation [96,97]. The simultaneous inhibition of these targets therefore leads to both reduced tumor vascularization and cancer cell death, and ultimately tumor shrinkage.

The evaluation of RCC patients demonstrated that treatment with sunitinib increased type-1 response including IFN-γ induction and reduction of MDSC [98] and Treg populations [99]. In murine models, treatment was also associated with decreased immunosuppressive effectors including surface PD-L1 and secretion of IL-10 [100]. Sunitinib decreased MDSC by promoting apoptosis of G-MDSC and decreased proliferation of M-MDSC [101]. When utilized in combination with immunotherapies including a dendritic cell (DC)-based vaccine [102], IL-12 and 4-IBB [100], or adoptive cell therapy [103], a more robust antitumor immune response, decreased tumor growth, and improved survival were observed in various murine models.

In treatment of glioma, there are conflicting reports of the efficacy of sunitinib in animal models. In murine models, sunitinib treatment did not improve survival when in combination with low dose radiotherapy [82] or another tyrosine inhibitor, gefintinib [83], but seemed to improve the efficacy of concurrent TMZ treatment [81].

The results in independent human clinical trials are also not promising. When administered as a single agent in multiple phase 2 trials in patients with malignant gliomas, no improvement was observed in either progression free survival or overall survival [84,85,86]. Many of these agents including sunitinib may have better efficacy when used in combination with immunotherapies as discussed earlier. The precise effects of sunitinib on MDSC populations in glioma and subsequent effects on immunotherapy remain to be determined. 

Other agents including chemotherapies, gemcitabine [104] and 5-flourouracil [105], have also been shown to reduce MDSC populations by induction of apoptosis while preserving T-cell functions. They have also demonstrated bolstered efficacy of tumor vaccines; and evaluations in glioma models are warranted. 

#### 2.2.4. Reducing MDSC Function

COX-2 expression and subsequent PGE_2_ production have been shown to induce immunosuppressive function in MDSC [106]. This pathway has been examined by our group in our *de novo* glioma model. Treatment of COX-2 inhibitors alone, including celecoxib can improve survival. PGE_2_ production was associated with increased CCL2, which can attract suppressive myeloid cells to the glioma site. Celecoxib treatment reduced CCL2 and increased CXCL10 production, recruiting CTL to the tumor site [37]. Similar results were observed in murine models of ovarian cancer, where COX-2 inhibition inhibited CXCR4 expression on MDSC and their responsiveness to CXCL12 [107].

Additionally, phosphodiesterase-5 (PDE-5) inhibitors, such as sidenafil, have been demonstrated to inhibit the function of MDSCs. In murine models of breast and colon cancers, treatment with sidenafil decreased arg1 and iNOS expression, resulting in increased numbers of tumor-infiltrating CD8+ T-cells [108]. Furthermore, combination of adoptive therapy with sidenafil therapy delayed tumor growth [108]. *In vitro* cultures with sidenafil improved T-cell functions in head and neck squamous cell carcinoma [108]. Recent reports from melanoma patients corroborate similar findings [109]. In glioma treatment, vardanefil, another PDE-5 inhibitor, when used in concert with adriamcyin, improved tumor tissue penetration by adriamcyin and survival [89]. The impact of myeloid modulation was not examined as a mediator of the improved survival observed in this study. 

#### 2.2.5. Tumor-Associated Macrophages/Microglia (TAM)

Human glioma exhibit extensive macrophages/microglia infiltrate, which can comprise up to 30% of the inflammatory cells at the tumor site [110]. Macrophages can be polarized to cytotoxic M1 or tolerogenic M2 type macrophages, depending upon the type of stimulation [67,111]. Lipopolysaccharides (LPS) and IFN-γ induce M1 type polarization, whereas IL-10 and TGF-β polarize them toward M2 type macrophages. Tumor-associated macrophages/microglia (TAM) encounter tumor-derived M2-inducing factors, and hence demonstrate M2 phenotype, which promotes tumor growth [67,111]. Although the relationship of these TAM to MDSC is not entirely clear, current evidence suggests that MDSC, particularly the LY6C^+^ monocytic subtype may be a progenitor to TAM, since these cells have been shown to be attracted to the tumor site, where they are induced into TAM-like cells by the tumor microenvironment [66]. TAM are enriched at the tumor site by recruitment via growth factors and chemokines including, CCL-2 [44,112,113], stromal-derived factor 1 (SDF-1) [114], S100 calcium binding protein B (S100B) [115], colony-stimulating factor-1 (CSF-1) [116] among others. In addition to recruitment, these factors have also been implicated in inducing tumor-promoting M2 phenotype so may prove to be important targets for glioma. In addition to immune suppression, M2 macrophages have been shown to produce matrix metalloproteinases (MMP), which promote glioma invasion. Additionally, TAM have been implicated in promoting tumor growth, angiogenesis, metastasis, and survival [117,118,119]. 

In gliomas, TAM may represent distinct lineages. Microglia are yolk sac derived CNS-resident macrophages, whereas macrophages are bone marrow derived cells [120]. Phenotypically, macrophages and microglia can be differentiated by CD45^Hi^ and CD45^Lo^ expression, respectively, in addition to CD11b expression [121]. The majority of the CD11b^+^ cells at the glioma site are CD45^Hi^, suggesting that bone marrow derived macrophages are the dominant population [121]. The distinct roles of these cells in tumor promotion and suppression remain currently unclear.

Both cell types in glioma exhibit M2 phenotype polarization, such as upregulation of CD163 and CD204, as well as via secretion of M2-associated cytokine including IL-4, IL-10, and TGF-β. M2 macrophages are associated with tumor progression and prevent the development of anti-tumor T cell response [111,112,113,114,115,116,117,118,119,120,121,122,123]. 

Macrophage numbers correlate with increasing histological grade, suggesting these cells may be promoting tumor progression [124]. Depletion of CD11b^+^ cells in gancyclovir treated CD11b-HSVTK mice exhibited improved survival and decreased tumor size [125], further demonstrating that the majority of tumor infiltrating CD11b^+^ cells represents a tumor-promoting population and subsequently that depletion of this population represents a potentially viable mechanism of promoting glioma rejection. In addition to depletion of TAM, another strategy to improve patient survival may be to inhibit M2 and concurrently promote M1 phenotype, which promotes anti-tumor cytotoxic activity [67,111]. This can be achieved by various strategies, including blockade of M2-associated effector mechanisms, inducing apoptosis and/or inhibiting recruitment of M2 to the tumor site, or polarization to an M1 phenotype. A more comprehensive review of modulating TAM in glioma can be found in [126]. 

CSF-1 recruits peripheral macrophages to the tumor site via CSF-1 receptor (CSF-1R) and is vital to induction of M2 phenotype. A recent study in pre-clinical GBM models showed that a specific inhibitor of CSF-1R did not deplete TAM but instead promoted their “re-education” via inhibition of M2-associated genes, resulting in hindered glioma growth and progression [116]. While a decrease in an M2-like gene signature was observed, besides IL-1β, no concurrent increase in M1-associated genes was observed with CSF-1R inhibition [116]. While CSF-1R inhibition appears promising for treatment of glioma, it remains determined whether abrogation of M2 phenotype with no increase in M1 function of TAM is sufficient to drive tumor rejection in patients with GBM. 

Additionally, many agents that target MDSC may also alter function of TAM. Future investigations should complement their analysis of immune cells by examining both MDSC and TAM in order to promote the development of effective strategies targeting glioma microenvironment. 

#### 2.2.6. Regulatory T-Cells (Tregs)

The significance of Tregs in limiting adaptive immune response and immunotherapy is becoming increasingly clear. CD4^+^FoxP3^+^ Treg population correlates with impairment of T-cell proliferation in peripheral blood specimens in GBM patients [127]. Moreover, tumor infiltration by Tregs correlates with tumor burden, as gliomas can alter properties of T-cells, resulting in increases of Treg compartments [128].

Glioma stem cells (GSC) can promote immunosuppression by secretion of immunosuppressive cytokines [129]. They have been shown to activate STAT3 and induce a Treg phenotype, which is further compounded by hypoxic conditions [130]. Glioma-derived factors have also been demonstrated to induce a Treg phenotype and suppressive functions [128]. A recent study in a murine model demonstrated that the majority of tumor-infiltrating Tregs are thymus derived as opposed to induced Tregs [131]. The differential role of induced Tregs and thymically-derived Tregs in glioma development requires further elucidation. 

CD4^+^FoxP3^+^ Tregs in gliomas have been shown to express a variety of immuno-regulatory molecules, such as CD25, CTLA-4, GITR (glucocorticoid-induced TNFR family related gene), and CXCR4 at high levels [77]. Indoleamine 2,3 dioxygenase (IDO) expression by tumor cells has been shown to inversely correlate with survival in GBM patients [132]. IDO expression was also shown to correlate with Treg infiltration into the glioma site as well as GITR expression by Tregs in mouse models. Accumulation and activation of CD4^+^FoxP3^+^ Tregs acts as a dominant immune escape mechanism for gliomas and underline the importance of controlling tumor-infiltrating Tregs in glioma immunotherapy. Treg numbers may be prognostic of vaccine therapy [133].

Multiple studies have examined the impact of depleting Tregs in bolstering anti-glioma immunity, the majority of these studies has utilized blocking anti-CD25 (IL-2Rα) mAb. In murine models, systemic administration of anti-CD25 mAb decreased the number of peripheral Tregs, inhibited their suppressive function and improved T-cell effector responses [76,134,135]. Depletion of Tregs alone improved the survival of mice bearing gliomas [76,134]. However, one study demonstrated that while Treg depletion improved tumor rejection, there was a lack of persistent immunological surveillance when mice were re-challenged with tumor cells [78]. In this study, the combination of Treg depletion and DC vaccine established more potent immune rejection as well as persistent anti-tumor immunity, highlighting the potential need of developing combination therapies [78]. 

In human clinical trials of anti-CD25 mAb (daclizumab) and EGFRvIII peptide vaccine, administration of daclizumab depleted circulating Tregs after a single dose, which did not return to the baseline until 120 days post administration. There were no significant changes to effector T-cell numbers. However, *in vitro* studies exhibited increased production of IFN-γ after daclizumab treatment. There was a correlation between increased humoral response to EGFRvIII with decreased Treg populations. The authors suggest that TMZ, a lymphodepleting agent, is potentially crucial to the selective depletion of Treg by daclizumab [79]. Studies in rats suggests that low dose TMZ may also preferentially deplete Tregs [80], and lead to decreased CCL2 production by glioma cells and inhibition of Treg recruitment [42]. The compounded depletion of Tregs by chemotherapies and depleting antibodies may be a potential way to promote immunotherapy efficacy. 

Other therapies for depletion of Tregs include cyclophosphamide [136], and other IL-2R targeting antibodies, such as IL-2 targeted toxins (denileukin difilitox) [137,138] and IL-2Rα-targeted immunotoxins (LMB-2) [139]. However, these approaches remain to be examined in glioma models or patients as potential methods for augmenting anti-tumor responses. There is a recent comprehensive review of these treatments that can be found here [140].

## 3. Vaccine Therapy for Gliomas

There are two major categories for cancer immunotherapy: active and passive immunotherapy. An active immunotherapy is a type of treatment that activates the immune system to take an active role in attacking cancer cells, such as cancer vaccines. On the other hand, passive immunotherapy approaches use special types of immune effector molecules or effector cells which are developed outside of a patient’s body. These include mAbs and adoptive cell transfer of autologous T-cells genetically engineered to attack tumor cells. Although passive immunotherapy approaches have recently made tremendous progresses, reviewed in [141,142], this review will focus on active immunotherapy approaches, especially clinical studies of vaccines in glioma patients. 

Recent randomized phase II–III studies in other cancer types have demonstrated therapeutic benefits of cancer vaccines [75,143], including the FDA approval for sipuleucel-T vaccine for castration-resistant prostate cancer [3], the first for non-virally induced cancers. These recent successes certainly spur the enthusiasm for development of effective vaccines for brain cancers, such as GBMs. Indeed, the history of developing brain tumor vaccines goes back to 1970s, when irradiated, autologous glioma cells were used for subcutaneous vaccinations [144]. 

Although there could be many ways to categorize the types of glioma vaccines, the current glioma vaccine approaches can be classified into two categories based on whether the vaccines are designed to target: (1) undefined, bulk glioma antigens or (2) molecularly defined antigen(s). There are advantages and possible disadvantages for each of the two approaches. The former will provide more diverse arrays of target-antigens, which may be critical to effectively treat antigenically heterogeneous tumors. However, those undefined antigens may be shared by normal brain cells, which may potentially lead to autoimmune encephalitis. While no verifiable cases of autoimmune encephalitis have been documented after active immunotherapy as of yet, there is a theoretical concern of inducing such a condition with use of checkpoint inhibitors as adjuncts to these therapies. On the other hand, vaccines targeting molecularly defined antigens allow more tumor-specific targeting of antigens that are uniquely or restrictedly expressed in glioma cells. Off-the-shelf formulation with one to several antigens may not address the heterogeneity of the individuals’ tumor antigenicity. Interestingly, a meta-analysis of 173 published peer-reviewed cancer vaccine trials found that patients vaccinated with whole-tumor antigen (n = 1,733) had a statistically higher rate of objective clinical responses than patients vaccinated with defined tumor antigens (n = 1,711; *p* < 0.0001) [145]. In the context of glioma vaccines, Prins *et al.* have compared the safety, feasibility, and immune responses of two DC vaccine formats; autologous tumor-lysate (ATL)-pulsed DCs and glioma-associated antigen (GAA) peptide-pulsed DCs [146]. In regard to feasibility, because of HLA subtype restrictions on the GAA-DC trial, only 6 of 15 screened patients were eligible for treatment, whereas 28 of 32 patients passed eligibility screening for the ATL-DC trial. A significant correlation was observed between decreased Treg ratios (post-vaccination/pre-vaccination) and overall survival (*p* = 0.004) in patients on both trials. Interestingly, Treg ratios were independently prognostic for overall survival in these patients, whereas tumor pathology was not in multivariate analyses. These data suggest a role of Treg ratios as a possibly universal biomarker in brain tumor vaccines [146]. 

### 3.1. Whole Glioma Antigen-Based Vaccines

Initial vaccination strategies for gliomas consisted of subcutaneous inoculations of irradiated, autologous [144] or allogeneic [147] glioma cells. This type of vaccine has the advantage of providing a panel of multiple potential GAAs that are naturally expressed by glioma cells. Especially, autologous glioma cells should allow immunizations against the most relevant GAAs expressed in the patient’s tumor (*i.e.*, tailored medicine). Potential downsides of this approach, however, include: (1) cumbersome procedures and quality assurance (QA)/quality control (QC) issues associated with large scale cultures of autologous glioma cells and (2) theoretical risks of autoimmune encephalomyelitis [144]. Nevertheless, this type of vaccine strategy has been carefully examined in GBM patients. 

Sloan *et al.* pioneered a combination approach of autologous GBM cell vaccine and adoptive transfer of *ex vivo* anti-CD3-stimualted T-cells [148]. The approach is currently being re-evaluated as phase I/II (NCT01081223) and phase II (NCT01290692) studies. Schneider *et al.* [149] and Steiner *et al.* [150] reported pilot clinical trials using autologous glioma cells modified with Newcastle-Disease-Virus, which is known to serve as an vaccine adjuvant to improve the efficacy of glioma vaccines. Ishikawa *et al.* reported a Phase I clinical trial using formalin-fixed glioma tissues as a source of antigens [151]. The advantage of this strategy is that formalin fixation preserves the specific antigenicity of glioma cells. No major adverse events were found in these studies.

Whole glioma cell-based vaccines have often employed peripheral blood monocyte-derived DCs as the vehicle of the vaccines. DCs are the most potent antigen presenting cells, driving the activation of T-cells in response to invading microorganisms [152]. The established culture methods of DCs from human peripheral blood monocytes has generated significant interest in using DCs in novel cancer vaccination strategies [152].

In early DC-based vaccine studies for GBMs, DCs were pulsed with either peptides eluted from autologous glioma cells or lysate of glioma cells. Yu *et al.* reported a phase I trial of vaccinations using DCs pulsed with peptides eluted from autologous glioma cells [153]. Later, Liau *et al.* also reported a phase I trial in patients with newly diagnosed GBM using DCs pulsed with acid-eluted glioma peptides [154]. In this study, the authors reported the median overall survival (OS) of 23.4 months and that the benefit of the vaccine treatment was more evident in the subgroup of patients with slowly-progressing tumors and in those with tumors expressing low levels of TGF-β2. A phase III, randomized study is currently ongoing with this approach (NCT00045968). 

However, pulsing DCs with eluted peptides requires a large culture of autologous glioma cells and time-consuming procedures, for which QA/QC is not always feasible. To overcome this issue, glioma cell lysate has been used to pulse DCs in a number of trials. Yamanaka *et al.* reported a phase I/II study using DC pulsed with glioma lysate. Patients received either DCs matured with OK-432 or DCs without OK-432-mediated maturation [155,156]. GBM patients receiving mature DCs had longer survival than those receiving DCs without OK-432-mediated maturation. Furthermore, patients receiving both intratumoral and intradermal DC administrations demonstrated longer overall survival than those with intradermal administrations alone [156]. Wheeler *et al.* reported another phase II clinical trial with lysate-pulsed DCs [157]. IFN-γ production levels from post-vaccine peripheral blood mononuclear cells (PBMC) correlated significantly with patients’ OS and time to progression. 

De Vleeschouwer *et al.* conducted a phase II DC vaccine study in pediatric and adult patients with recurrent GBM [158]. DCs were loaded with autologous tumor lysate and matured with TNF-α, IL-1β as well as PGE2 and injected intradermally. Total resection was a predictor for better progression-free survival (PFS) both in univariable analysis and after correction for the other covariates. For OS, younger age and total resection were predictors of a better outcome in univariable analysis but not in multivariable analysis. The importance of age and a minimal residual disease status at the start of the vaccination is underscored. The same group subsequently conducted a phase I/II study of lysate-loaded DC vaccines in newly diagnosed patients, whose tumors were maximally resected and undergo standard-of-care chemo-radiation therapy [159]. These patients were administered DC vaccines weekly for 4 times after chemo-radiation therapy. Boost vaccines with lysates were given during the adjuvant chemotherapy with TMZ. PFS at six months was 75%. Median OS for all patients was 24 months (range: 13–44 months). The only serious adverse event was one case of ischemic stroke eight months postoperatively [159]. 

Prins *et al.* performed a phase I dose-escalation study of DC vaccines loaded with autologous tumor lysate in combination with a toll-like receptor (TLR) agonist, either imiquimod or poly:ICLC, as an immunoadjuvant in adult newly diagnosed (N = 15) or recurrent (N = 8) GBM [160]. T-cell infiltration was increased after vaccination in tumors resected or biopsied at recurrence; and this increase was associated with the mesenchymal gene expression signature. There were no grade 3 or 4 adverse events. Interestingly, patients with the mesenchymal gene expression signature (n = 9) had significantly better survival than a randomly selected control mesenchymal group (n = 82), whereas no difference was seen in patients with the proneural signature. This approach, combining lysate DC vaccines and TLR agonists, is being evaluated as a phase II study comparing relative immunological activities of imiquinod, resiquimod or poly:ICLC (NCT01204684). A group in University of Miami is also opening a phase I study combining DC vaccines and imiquimod application in high-grade glioma patients (NCT01808820). 

Fadul *et al.* focused on the immune response, PFS, and OS of newly diagnosed GBM patients treated with an intranodal autologous tumor lysate DC vaccination [161]. Tumor-specific CTLs correlated with both PFS and OS. All patients survived past 6 months post-diagnosis and a progression-free survival of 9.5 months was reported. Median OS was 28 months, which is significantly higher than the OS of 18–21 months in the matched historical control data.

Although these phase II studies all demonstrate preliminary promising signs of clinical activity, each of the vaccine platforms should ultimately be evaluated in randomized phase II or III studies. Cho *et al.* conducted a phase II randomized study of autologous GBM-lysate-loaded DC vaccines [162] in newly diagnosed GBM patients who also receive standard-of-care chemo-radiation therapy. Eighteen patients underwent conventional treatment (surgery, radiotherapy, and chemotherapy) and received adjuvant autologous DC vaccines, and 16 patients (control group) underwent conventional treatment only. The median OS for the vaccine group was 31.9 months and for the control group was 15.0 months (*p* < 0.002). Although the sample size is relatively small, these data strongly suggest a clinical activity of autologous GBM-lysate-loaded DC vaccines. Randomized phase II studies evaluating the clinical efficacy of lysate-loaded DCs are also being conducted in Austria (NCT01213407) and China (NCT01567202). 

Alternative to autologous tumor-loaded DC-based vaccines Crane *et al.* recently reported their results of a phase I study evaluating feasibility, safety and immunological activities of a 96 kD chaperone heat-shock protein (HSP-96) derived from surgically resected recurrent GBM. Of 28 patients screened, 16 patients did not receive any vaccine, including nine patients from whom inadequate amounts of tumor tissue were obtained for vaccine production. Nonetheless, 11 of 12 vaccine-recipients with positive peripheral blood responses against the vaccine showed a median survival of 47 weeks after surgery and vaccination, compared to 16 weeks for the single non-responder. Phase II multi-center studies are underway in newly diagnosed (NCT00905060) and recurrent (NCT00293423) GBM patients. As a novel approach targeting the whole GBM-derived antigen, DC vaccines loaded with autologous brain tumor stem cell-derived mRNA are currently being evaluated for their feasibility and safety at Duke University (NCT00890032) and Oslo University (NCT00846456) as two independent projects.

### 3.2. Vaccines Targeting Glioma-Associated Antigens (GAAs)

In contrast to the whole GBM antigen-based vaccine approaches, vaccines targeting molecularly defined GAAs are less of a concern for autoimmunity and provide “off the shelf” feasibility. A wide range of peptide-based vaccines have been evaluated. Izumoto *et al.* reported a Phase II clinical trial using a single Wilms’ tumor gene product (WT1)-derived peptide [163]. In this study, they reported a median PFS of 20 weeks and a possible association between the WT1 expression levels and clinical responses. An apoptosis inhibitor protein survivin has been found to be expressed in nearly 100% of gliomas, but not in normal brain tissues [164,165]. Ciesielski *et al.* have developed a novel, modified Survivin epitope SVN53-67/M57. This epitope elicits multi-epitope CTL and helper T-cell responses in a non-HLAA2 restricted manner against glioma cells that display wild-type survivin epitopes [166]. To determine the toxicity profile of the SVN53-67/M57-KLH peptide in montanide ISA 51 plus with GM-CSF, a phase I study is underway (NCT01250470).

While GAAs are defined as antigens that are expressed in glioma cells in a relatively restricted manner, the ideal vaccine should target truly tumor-specific antigens to assure the lack of autoimmunity. Among the variants of EGFR, EGFRvIII is the most commonly observed in human tumors (reviewed in [167,168,169,170,171]). This protein results from the in-frame deletion of exons 2–7 and the generation of a novel glycine residue at the junction of exons 1 and 8. This novel juxtaposition of amino acids within the extra-cellular domain of the EGF receptor creates a tumor-specific and immunogenic epitope. EGFRvIII expression has been seen in many types of human cancers including GBM, breast adenocarcinoma, non-small cell lung carcinoma, ovarian adenocarcinoma, and prostate cancer, but is rarely observed in normal tissue, making it ideal as both a diagnostic marker and a target for immunotherapy. EGFRvIII is expressed in 24% to 67% of GBM cases, and in patients surviving > or =1 year, the expression of EGFRvIII is an independent negative prognostic indicator [172,173]. Sampson *et al*. recently reported a Phase II study targeting the EGFRvIII epitope in newly diagnosed GBM patients who received gross total resection [174]. This study reported a median PFS of 14.2 months and a median OS of 26.0 months. In addition, they identified that the development of specific antibody or delayed-type hypersensitivity responses to EGFRvIII significantly correlated with the OS. Interestingly, recurrent GBMs following the vaccines did not express EGFRvIII, indicating the outgrowth of antigen-loss variants of the GBM cells. Randomized studies are underway to determine the activity of this approach in newly diagnosed (NCT01480479) and recurrent (NCT01498328) GBMs as phase III and II studies, respectively. Also, as an extension of this regimen, a new approach combining the EGFRvIII-targeted vaccine and anti-CD25 mAbs, which are designed to deplete regulatory T-cells, is being evaluated in a phase I study (NCT00626015). 

When single or oligo antigens are selected and targeted by vaccines, it also seems necessary to diversify the response to wider arrays of GAAs, and harness the concepts of epitope spreading to address the problems of tumor immune escape, while avoiding the augmentation of deleterious CNS autoimmune responses [175]. An ideal GBM vaccine therefore should be targeting multiple GAAs that can be recognized by individual patients’ immune system as immunogenic antigens. 

Yajima *et al*. reported a phase I study of peptide-based vaccinations in patients with recurrent malignant gliomas [176]. In this study, prior to the first vaccine, each patient’s PBMCs were evaluated *in vitro* for cellular and humoral responses against a panel of antigens, and peptides that induced a positive response were used for vaccinations. The regimen was well tolerated and resulted in an 89-week median survival of treated patients. However, there is little evidence that the antigens used in this study are expressed in gliomas at high levels. More recently, as an extension of the approach, Terasaki *et al.* reported a phase I trial using 14 HLA-A24-binding peptides [177]. This study evaluated immune responses with dose escalation of peptides and defined 3 mg/peptide as the phase II-recommended dose. 

Phuphanich *et al.* performed a phase I study evaluating the safety and immune responses to an autologous DC vaccine pulsed with class I peptides from GAAs expressed on gliomas and overexpressed in their cancer stem cell population [178]. The authors targeted HLA-A1- or A2-binding epitopes derived from HER2, TRP-2, gp100, MAGE-1, IL13Rα2, and AIM-2. They observed correlations of increased PFS and OS with quantitative expression of melanoma-associated antigen (MAGE)1 and absent in melanoma (AIM)-2. Interestingly, five patients who underwent a second resection for recurrent tumors showed decrease or absence in expression levels of a GBM stem cell marker, CD133. Median PFS in newly diagnosed patients was 16.9 months, and median OS was 38.4 months. A phase II, randomized, double-blind, controlled-phase IIb study with this approach is underway (NCT01280552). 

In our laboratory, based on our preclinical data demonstrating that type-1 CTLs are capable of mediating effective anti-CNS tumor immunity [179,180,181,182], we have completed a phase I/II study of vaccines evaluating safety and immunological activities of vaccines using α-type-1-polarized DCs (αDC1) that are able to produce high levels of IL-12 and induce long-lived type-1 T-cell responses [183]. In this study, patients with recurrent malignant glioma received intra-lymphnodal injection of αDC1 loaded with synthetic peptides for GAA epitopes and administration of poly:ICLC in HLA-A2^+^ patients with recurrent malignant gliomas. GAAs for these peptides are EphA2, IL-13Rα2, YKL-40, and gp100. The regimen was well-tolerated and induced positive immune responses against at least one of the vaccination-targeted GAAs in peripheral blood mononuclear cells in 83% of patients. Peripheral blood samples demonstrated significant upregulation of type 1 cytokines and chemokines, including interferon-α and CXCL10. For at least 12 months, nine patients achieved progression-free status. Two patients (one with recurrent GBM and one with recurrent anaplastic astrocytoma) demonstrated a sustained complete response. IL-12 production levels by αDC1 positively correlated with time to progression. These data support safety, immunogenicity, and preliminary clinical activity of poly:ICLC-boosted αDC1-based vaccines and warrant further development of this approach. With regard to the use of IL-13Rα2HLA-derived class I epitopes, Iwami *et al.* have demonstrated positive T-cell responses against an HLA-A24-binding IL-13Rα2-derived epitope in their phase I vaccine study in patients with malignant glioma [184].

Recently, Dutoit *et al.* have identified an attractive class of HLA-A2-binding epitopes that are naturally presented in HLA-A2 molecules of GBM tissues [185]. They eluted >3,000 HLA-A2-restricted peptides from fresh GBM tumor tissues. They prioritized further investigation of 10 GBM-associated antigens based on high expression in tumors, very low or absent expression in healthy tissues, implication in gliomagenesis and immunogenicity. Patients with GBM showed no T cell tolerance to these peptides, and patients’ CD8+ T cells stimulated with these peptides demonstrated specific lysis of GBM cells. Furthermore, antigen-specific CD8+ T cells were present in GBM tissues. The peptides identified in this study are currently being evaluated as phase I studies in newly diagnosed GBM patients through Cancer Research UK (ClinicalTrials.gov Identifier: NCT01222221) and the National Cancer Institute (NCT01403285). 

In addition to these vaccine strategies administered peripherally, several types of viral therapies, most of which are administered directly to the intracranial tumor site, can also be considered immunotherapy, as they are expected to cause an immune response as a part of the therapeutic mechanisms (e.g., NCT01814813 and NCT00751270). 

## 4. Applications of Immunotherapy for Pediatric Brain Tumors

In comparison to the extensive research that has been conducted on immunotherapy for adult brain tumors, the number of pediatric immunotherapy studies is relatively modest. However, significant recent interest has been focused on the use of vaccination or immunomodulation in high-risk tumors, such as brainstem gliomas and high-grade non-brainstem gliomas, as well as recurrent tumors, such as low-grade gliomas and ependymomas. Such approaches are particularly promising in the pediatric age group since affected patients are likely to have intact immunity. Recent pilot studies of immunotherapy for pediatric malignant brain tumors have suggested that children may exhibit a productive immunological response as well as clinical activity [158,186,187,188,189], although these small pilot studies were primarily designed to assess safety, and conclusions of the clinical efficacy are inferential, at best. 

Most studies to date, including several that are ongoing (e.g., NCT01326104), have involved administration of tumor lysate or tumor RNA with DC [158,186,187]. As noted earlier, these approaches have the advantage of exposing the immune system to a host of tumor-related antigens, but the disadvantage is that *ex vivo* manipulation is required and that substantial amounts of tumor are required as the vaccine source, which may be a limiting factor for children with deep-seated tumors, such as diffuse brainstem gliomas where biopsies, if performed, are generally quite small. An alternative approach, using an allogeneic tumor cell lysate, has also been proposed (NCT01400672) for lesions, such as brainstem gliomas, that do not have readily accessible tumor material.

As an additional strategy, a handful of recent studies have focused on peptide-based vaccination approaches, including NCT01058850, which administers an EGFRvIII peptide; and NCT00935545, which administers a multipeptide cocktail, both of which are in progress. 

An ongoing study at our institution combines subcutaneous vaccinations with peptides for GAA epitopes emulsified in Montanide-ISA-51 given every 3 weeks for eight courses along with intramuscular injections of an immunoadjuvant, poly-ICLC, in HLA-A2+ children with newly diagnosed brainstem gliomas (BSG), high-grade gliomas (HGG), or recurrent gliomas [188,189]. The GAAs targeted in this trial are EphA2, IL13Rα2, and survivin. Among 33 patients enrolled through June 30, 2012 (to allow a minimal follow-up duration of at least one year for toxicity monitoring or until disease progression or off-study criteria were met); 14 had previously untreated BSG and received irradiation alone, six had newly diagnosed BSG treated with irradiation and concurrent chemotherapy, four had recurrent high-grade glioma, four had newly diagnosed high-grade glioma treated with irradiation and concurrent chemotherapy, and five had multiple recurrent low-grade glioma, after failure of numerous prior regimens. The primary objectives of this study were to assess safety, given that at the time it was launched, it this was the first such “peptide + adjuvant” vaccine trial for pediatric brain tumors. 

Principal toxicities have included local injection site reactions and low grade fevers and flu-like symptoms in almost all patients, which have been generally mild, as well as several instances of possible immunologically-mediated pseudoprogression. Both clinical and immunological responses have been observed, although formal outcome analysis is pending, since patients remain on study. A multi-institutional trial of this GAA cocktail in conjunction with the immunoadjuvants Imiquimod [160,190] and GM-CSF is planned within the Pediatric Brain Tumor Consortium. 

In addition to the above approaches, recent interest has been directed at the implementation of approaches targeting immune checkpoint inhibitors, such as ipilumimab and anti-PD1. The advancement of such studies is awaiting preliminary safety data in adult patients with brain tumors.

## 5. Conclusions

We reviewed recent progresses in the field of brain tumor immunology with particular foci in the area of immune-checkpoint mechanisms and development of active immunotherapy strategies. In the last decade, a number of novel immunoregulatory mechanisms have been identified and characterized, and most of them seem to mediate immunosuppression in GBM patients. Promising news is that specific mAbs designed to block each of those “checkpoints” have been developed and show efficacy in other cancers, such as melanoma. The main reason we focused our review in these areas is because we believe that development of active immunotherapy approaches should integrate those checkpoint-blockade agents to enhance the efficacy of therapeutic approaches. With these agents available, it is going to be quite an exciting time in the field. Nonetheless, there are inherent challenges in combinatory approaches. For example, when each of these agents is owned by separate industries with intellectual property, creative combinatorial strategies may not be implemented as efficiently as one would expect. In addition, we need to address whether the mode of delivery (*i.e.*, local *vs.* systemic) significantly impacts the safety and efficacy of antibody- and compound-based approaches if the target is within the brain. The eventual success of immunotherapies for brain tumors will be dependent upon not only an in-depth understanding of immunology behind the brain and brain tumors, but also collaboration and teamwork for the development of novel trials that address multiple layers of immunological challenges in gliomas.
